# Breeding honey bees (*Apis mellifera* L.) for low and high *Varroa destructor* population growth: Gene expression of bees performing grooming behavior

**DOI:** 10.3389/finsc.2023.951447

**Published:** 2023-03-06

**Authors:** Nuria Morfin, Brock A. Harpur, Alvaro De la Mora, Ernesto Guzman-Novoa

**Affiliations:** ^1^ British Columbia Technology Transfer Program, British Columbia Honey Producers Association, Victoria, BC, Canada; ^2^ Department of Biochemistry & Molecular Biology, The University of British Columbia, Vancouver, BC, Canada; ^3^ Department of Entomology, Purdue University, West Lafayette, IN, United States; ^4^ School of Environmental Sciences, University of Guelph, Guelph, ON, Canada

**Keywords:** *Apis mellifera*, honey bees, *Varroa destructor*, viruses, selective breeding, grooming, RNA sequencing

## Abstract

**Introduction:**

Social organisms, including honey bees (*Apis mellifera* L.), have defense mechanisms to control the multiplication and transmission of parasites and pathogens within their colonies. Self-grooming, a mechanism of behavioral immunity, seems to contribute to restrain the population growth of the ectoparasitic mite *Varroa destructor* in honey bee colonies. Because *V. destructor* is the most damaging parasite of honey bees, breeding them for resistance against the mite is a high priority of the beekeeping industry.

**Methods:**

A bidirectional breeding program to select honey bee colonies with low and high *V. destructor* population growth (LVG and HVG, respectively) was conducted. Having high and low lines of bees allowed the study of genetic mechanisms underlying self-grooming behavior between the extreme genotypes. Worker bees were classified into two categories: ‘light groomers’ and ‘intense groomers’. The brains of bees from the different categories (LVG-intense, LVG-light, HVG-intense, and HVG-light) were used for gene expression and viral quantification analyses. Differentially expressed genes (DEGs) associated with the LVG and HVG lines were identified.

**Results:**

Four odorant-binding proteins and a gustatory receptor were identified as differentially expressed genes. A functional enrichment analysis showed 19 enriched pathways from a list of 219 down-regulated DEGs in HVG bees, including the Kyoto Encyclopedia of Genes and Genomes (KEGG) term of oxidative phosphorylation. Additionally, bees from the LVG line showed lower levels of *Apis rhabdovirus* 1 and 2, *Varroa destructor virus* -1 (VDV-1/DWV-B), and *Deformed wing virus-A* (DWV-A) compared to bees of the HVG line. The difference in expression of odorant-binding protein genes and a gustatory receptor between bee lines suggests a possible link between them and the perception of irritants to trigger rapid self-grooming instances that require the activation of energy metabolic pathways.

**Discussion:**

These results provide new insights on the molecular mechanisms involved in honey bee grooming behavior. Differences in viral levels in the brains of LVG and HVG bees showed the importance of investigating the pathogenicity and potential impacts of neurotropic viruses on behavioral immunity. The results of this study advance the understanding of a trait used for selective breeding, self-grooming, and the potential of using genomic assisted selection to improve breeding programs.

## 1 Introduction

Eusociality is a major evolutionary transition in the history of life on earth (1, 2). It represents the evolution of a new unit of selection: the superorganism. Eusociality has evolved dozens of times independently across the Animalia but it has often occurred within the Hymenoptera (e.g. bees, wasps, and ants) (3). Despite some advantages of sociality, group-living in eusocial colonies may increase the risks of infectious diseases (4–6). Parasites and pathogens can reduce their host fitness, and thus, natural selection would favor traits that could help the host avoid or reduce the harm caused by pathogens and parasites (5, 7). In some cases, social species evolve novel forms of defense to help ward off infection (5). Social insects for example, use a variety of behavioral mechanisms to avoid infections, reduce parasite loads, or reduce pathogen transmission between nestmates (5, 7).

Honey bees have shown to be a good model organism to investigate social defense mechanisms and host-pathogen interactions (8). The spillover of the parasitic mite *Varroa destructor* from its original host, the Asian honey bee, *Apis cerana*, to the western honey bee, *Apis mellifera*, gives us a unique opportunity to study behavioral immune responses against the ectoparasite in its non-adapted host (9). Interestingly, western honey bees use some of the same defense mechanisms expressed by the Asian bee to reduce the impact of *V. destructor*, including allogrooming, self-grooming, and hygienic behavior, although to a lesser extent than *A. cerana*, and thus, *A. mellifera* remains highly vulnerable to the parasitosis.

It is important to better understand defense mechanisms of the western honey bee to control *V. destructor*, as its parasitism is directly linked to extreme colony losses in North America and around the world, which severely impacts agribusinesses, including the beekeeping industry and bee pollinated crops (10–12). One approach to control the damage caused by *V. destructor* is to select bees resistant to the parasite (13). Using honey bee genotypes that are resistant to *V. destructor*, beekeepers could decrease the dependence on synthetic acaricides by incorporating an Integrated Pest Management strategy, which would help reduce the damage caused by the parasite and the viral infections associated with it, like those caused by *Deformed wing virus-A* (DWV-A) and *Israeli acute paralysis virus* (IAPV) (14–16).

There are some traits that honey bee breeders could select for in their stocks to increase their resistance to *V. destructor*. One such trait is grooming behavior, by which bees, using their mandibles and legs, remove *V. destructor* mites from their bodies, sometimes mutilating them (17). Grooming behavior, and the proportion and severity of mite mutilations, have been found to be associated with increased colony survival in beekeeping operations (18, 19). Because of its apparent effect on colony survival and because it is a heritable trait (20–23), selection for grooming behavior has been incorporated into several bee breeding programs in North America (18, 19, 22). While selecting for this trait seems to increase the resistance of honey bee stocks to *V. destructor*, there are two major challenges to incorporate grooming behavior assessments into breeding programs: 1) Phenotyping is often labor-intensive: a typical mite-drop assay requires at least two days of work in the field and additional time on a microscopy assessment to score the severity of mite mutilations, and 2) Phenotyping needs to be performed on full sized colonies: it can take months for a colony to develop to full size. Additionally, a successful breeding program would need to quantify grooming in hundreds of colonies over the course of several years to identify colonies with low and high proportion of groomers (18, 19). Thus, a phenotypic selection for grooming behavior in the field can be challenging. A better understanding of the molecular mechanisms behind social immune responses, like self-grooming, will provide tools to assist breeding programs by incorporating genomic selection approaches. Genomic selection has proven to be superior at predicting traits of interest in untested lines compared to conventional phenotypic frameworks, thus, increasing the efficiency of the selection programs (24).

Two genotypes of honey bees are being selected at the Honey Bee Research Centre, University of Guelph (ON, Canada): one for low *V. destructor* population growth (LVG) and one for high *V. destructor* population growth (HVG) (25). *Varroa destructor* population growth has been associated with grooming behavior and the intensity with which bees groom (26). Worker bees from LVG colonies tend to groom more intensively, compared to bees from unselected colonies (26). The LVG and HVG populations we developed provide a unique opportunity to study the genetic differences associated with *V. destructor* loads, the viruses the mite transmits, and specifically grooming. This is important, because the aims of a selective breeding program should not only be to develop resistant honey bee stock, but to also understand the molecular basis of honey bee resistance to *V. destructor* parasitism and to develop molecular tools to improve selection.

To gain knowledge about the molecular basis of resistance to *V. destructor*, we used self-grooming assays to classify worker bees of the above selected genotypes into two categories: ‘light groomers’ and ‘intense groomers’ (26, 27). The brains of bees from the different categories (LVG-intense, LVG-light, HVG-intense, and HVG-light) were used for RNA extraction, RNA sequencing (RNA-seq), gene expression analysis, and viral identification and abundance. Here we present results of differentially expressed genes (DEGs) and enriched pathways for LVG and HVG bee genotypes, as well as for grooming intensity and viral analyses, which adds to our understanding of the molecular mechanism behind behavioral immune responses.

## 2 Methods

### 2.1 Self-grooming assays

The selection process of LVG and HVG genotypes consisted of assessing more than 300 honey bee colonies with queens of different genetic backgrounds (generation 0, 2018). The colonies were evaluated in spring and summer for fallen mites using sticky papers placed on the bottom boards of the hives (19, 25). The colonies with the highest proportional increase of mites were designated HVG, and the six colonies with the lowest proportional increase of mites were designated LVG. Three colonies of each genotype were used to produce a new generation of 150 colonies the following two years (generations 1 and 2, respectively). Worker bees from LVG and HVG colonies of the second generation were used for self-grooming assays (25). In generation 2, LVG colonies showed a 1.7 fold mite population growth increase, which was six time lower compared to the 9.6 fold mite increase of the HVG colonies (25).

A total of 2,499 worker bees were collected from three colonies selected for LVG, and from three colonies selected for HVG at the Honey Bee Research Centre, University of Guelph (43° 32’ 11.292”N, -80° 12’ 50.9898”W) (25). Briefly, for each colony, three frames with nest bees from the brood chamber were shaken into a 5 L plastic container, and a scoop of them was collected and transported into the lab. We used nest bees to assess self-grooming behavior as they have been reliably assayed for this behavior in the past (26, 28). The classification system for grooming bees was based on previous studies which identified a higher proportion of intense groomers from colonies of presumably resistant genotypes to *V. destructor* parasitism (26, 28), and differences in gene expression and lipidome profile between bees grooming lightly and intensively (27, 29). Each individual worker bee was placed inside a Petri dish (100 mm x 15 mm; Fisher Scientific, Mississauga, ON, Canada) covered with a perforated lid, and was left there for 2 min to become used to the environment. After that, approximately 20 mg of wheat flour (Robin Hood^®^, Markham, ON, Canada) was put on her thorax using a fine paint brush (6 mm x 11 mm; DeSerres^®^, Oakville, ON, Canada) to stimulate grooming instances. Wheat flour is a reliable proxy of *V. destructor* irritation as previously demonstrated (28). Each bee was observed for 3 min and the time of first response to the stimulus as well as the intensity with which the worker bee removed the flour by grooming was recorded by one observer. The observer was blind to the genotypes. The self-grooming behavior of bees was assessed based on motor response to the irritant, specifically the number of legs used to groom and the intensity of their body movements. Bees were classified as ‘light’ groomers if slow movements were noted and no more than two legs were used to remove the irritant, or as ‘intense groomers’, if vigorous shaking and wiping was observed and if the bee used three or more legs to remove the irritant. Bees that showed intermediate expressions of self-grooming behavior were discarded to prevent misrepresentation of the behaviors, and make sure we were selecting for extreme behavioral responses (light and intense). To record time of first grooming, the observer had a stopwatch with a resolution of 1/100 s and an accuracy of 0.001% (Fisherbrand, Mississauga, ON, Canada), the stopwatch started when the flour was placed on top of the bee’s body. After the self-grooming trials, each bee was flash frozen on dry ice.

### 2.2 Brain dissections, RNA extraction and RNA-seq

**Figure 1:**
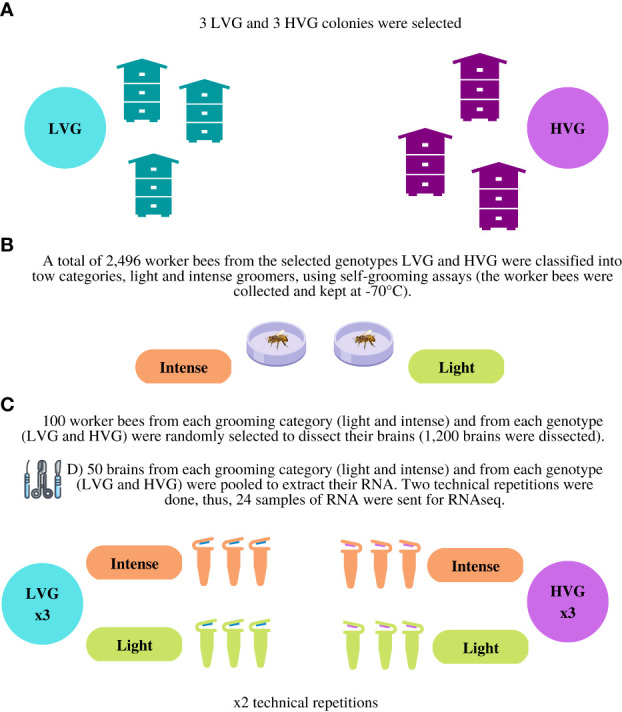
Methodology to obtain samples of RNA for RNA-seq analysis. Description: **(A)** Three LVG and three HVG colonies were selected for the study. **(B)** A total of 2,496 worker bees from the selected lines (LVG and HVG) were classified into two categories, light and intense groomers, using self-grooming assays (the worker bees were collected and kept at -70°C). **(C)** One hundred worker bees from each grooming category (HVG-intense, HVG-light, LVG-intense, LVG-light) were randomly selected to dissect their brains (1,200 brains were dissected). **(D)** Fifty brains from each category (HVG-intense, HVG-light, LVG-intense, LVG-light) were pooled to extract their RNA and two technical repetitions were done. Thus, 24 samples of RNA were sent for RNA-seq.

The brains of 50 randomly selected worker bees from each category (HVG-intense, HVG-light, LVG-intense, LVG-light) were pooled to extract RNA. There were three biological repetitions (three colonies of each genotype) and two technical replicates totaling 24 RNA extractions of pooled brains and 1,200 dissections (each of the 24 RNA samples consisted of the pooled RNA of 50 brains; performed as per Morfin et al. (29) (**Figure 1**). Total RNA was extracted using TRIzol™ (Invitrogen, California, USA) following the manufacturer’s instructions. A spectrophotometer was used to determine the absorbance ratio of the RNA; values between 1.8 and 2.0 for 260/280 nm and values between 2.0 and 2.2 for 260/230 nm were considered acceptable for purity. The samples were kept at -70°C until sequencing.

A total of 24 RNA samples were sent to McGill University (Génome Québec Innovation Centre, Montreal, QC, Canada) to perform a high throughput sequencing analysis. A second quality assessment of the RNA was done using a Bioanalyzer prior to cDNA library construction with NEB kit Illumina (San Diego, CA, USA). RNA sequencing was performed as 150 bp paired end reads using NovaSeq 6000 S4 (Illumina, San Diego, CA, USA).

### 2.3 Statistical analyses and design

Fisher exact tests of independence were used to determine significant differences in the proportion of LVG and HVG bees performing intense or light grooming. The same tests were used to determine significant differences in the proportion of DEGs in different pairwise comparisons (LVG-light vs HVG-intense, LVG-light vs HVG-light, LVG-intense vs HVG-light, LVG-intense vs HVG-intense, and HVG-light vs HVG-intense), and adjusted residuals were calculated for *post hoc* analyses. As the data was not normally distributed, based on Shapiro Wilk test, a Kruskal-Wallis and Conover-Iman procedures were used to compare the time of first grooming between honey bees classified as HVG-intense, HVG-light, LVG-intense, and LVG-light. Additionally, an Aligned Rank Test ANOVA (ART) was conducted to determine the interaction between genotype (LVG and HVG) and grooming intensity (intense or light grooming) on the time of first response to the irritant.

The gene expression analysis was done using the Kallisto/Sleuth pipeline as per Waterhouse et al. (30). Briefly, FastQC was used to assess the quality of the raw sequence data (31). A transcriptome index based on the latest honey bee transcriptome [Amel_HAV3.1 genome build; (32)] was built with Kallisto (v 0.11.0; with a 100 bootstrap value) (33, 34).The output produced by Kallisto was processed using Sleuth (v 0.30.0) and Shiny (v 1.6.0) within R (v 4.1.0) (35–37). Normalization was done automatically using transcript per million (TPM) values generated by Kallisto in Sleuth (34, 35). To conduct a gene-level analyses with Sleuth, the R BioMart package (v 2.49.1) was used to match gene names to transcripts (38). A functional enrichment analysis was done with g:Profiler (39), using cumulative hypergeometric test to evaluate the functional enrichment of the gene list and perform multiple test corrections with g:SCS (set counts and sizes). Additionally, a mixed factorial analysis was used to correlate the quantitative variables time of first grooming (mean of time of first grooming at a colony level; **Tables 1**; **S1**) and transcript expression (TPM), to the qualitative variables varroa growth (HVG and LVG) and grooming intensity (intense and light). Lastly, a Pearson correlation test was conducted to determine the association between TPM and time of first grooming in odorant binding proteins 16, 17, 18, 21, and the gustatory receptor 10.

**Table 1:** Time of first grooming response (s) from bees of each experimental colony and category (LVG-intense, LVG-light, HVG-intense, and HVG-light).

Colony ID	Category	Mean (s)	± SEM
478	LVG-intense	2.86	0.44
478	LVG-light	3.20	0.55
499	LVG-intense	6.84	0.49
499	LVG-light	11.41	0.77
113B	LVG-intense	3.46	0.27
113B	LVG-light	4.66	0.61
111A	HVG-intense	6.13	0.35
111A	HVG-light	11.95	0.88
114A	HVG-intense	5.16	0.36
114A	HVG-light	12.40	0.91
338	HVG-intense	4.74	0.28
338	HVG-light	12.56	0.88

Mean (± SEM) first grooming response time (s) of bees from HVG and LVG selected colonies that self-groomed lightly or intensively within 3 min after placing 20 mg of flour on their thoraces.

The FastVirome pipeline was used to identify and quantify viral transcript abundance (40), with a precomputed Kallisto index containing the sequences of 20 viruses known to infect honey bees (33), retrieved from the National Center for Biotechnology Information (41). Degust was used to compare the viral transcript abundance between bees from LVG and HVG colonies (42). Lastly, a Pearson correlation test was applied at a colony level to determine the correlation between the time of first grooming of bees in different categories (LVG-intense, LVG-light, HVG-intense, and HVG-light), viral abundance (*Apis rhabdovirus* 1 and 2, ABPV, BQCV, KBV, DWV, VDV-1 and 2, and *Apis filamentous virus*), and transcript abundance of the 23 significantly DEGs (p<0.05). Statistical analyses were performed using R version 3.5.3 and XLSTAT 2020 with the significance level set at p<0.05 (α of 0.05). Only for Pearson correlation tests an α of 0.1 was reported (37, 43).

## 3 Results

**Figure 2:**
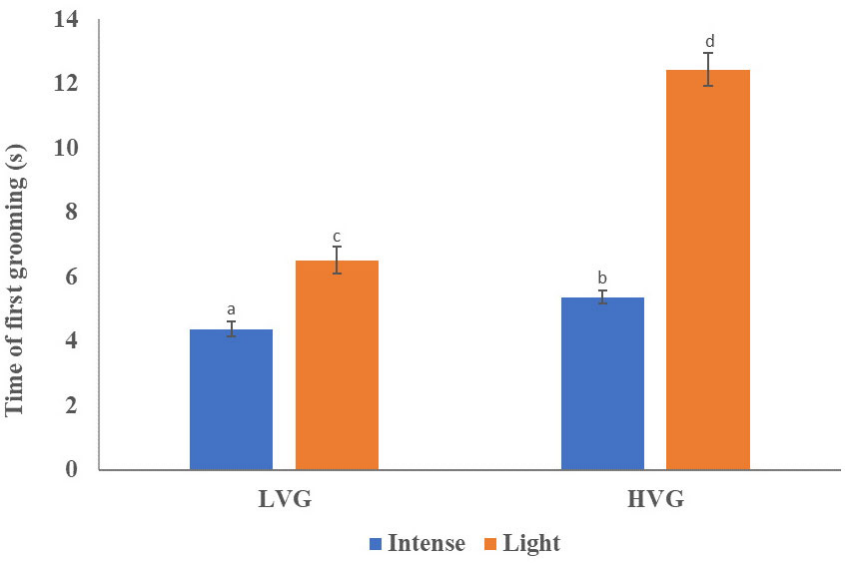
Time of first grooming response. Mean (± SEM) first grooming response time (s) of bees from HVG and LVG selected colonies that self-groomed lightly or intensively within 3 min after placing 20 mg of flour on their thoraces. Bars with different letters above them represent significant differences based on Kruskal-Wallis tests and Conover-Iman procedures (p<0.05). Non-ranked data is presented.

Time to first grooming varied between light and intense bees from LVG and HVG colonies There was a significantly higher proportion of intense groomers than light groomers in bees from both genotypes ( $\chi_{(1,2499)}^{2}$ = 3.84, p=0.002; **Table 2** and **Table S1**). Also, significant differences in the time of first grooming were found (F_(3)_=7.81, p<0.0001; **Figure 2**; **Tables 1**, **S1**). LVG-intense groomers responded significantly faster to the irritant (4.38 ± 0.23 s; p<0.0001), followed by HVG-intense (5.34 ± 0.19 s), LVG-light (6.42 ± 0.41 s), and HVG-light (12.30 ± 0.51 s) groomers. Additionally, no interaction between genotype (LVG or HVG) and grooming intensity (intense or light) was found (F_(1,3)_= 2.51, p=0.11) and no effect of the genotype (F_(1,3)_=2.08, p=0.14), but a significant effect of the intensity of self-grooming was observed (F_(1,3)_=5.86, p=0.0028), supporting that intense groomers were faster at responding to the irritant compared to light groomers.

**Table 2:** Number of LVG and HVG bees grooming lightly and intensively.

Genotype	Number of bees that groomed lightly	Number of bees that groomed intensively	Proportion of bees that groomed intensively
LVG	461	690	0.59
HVG	458	887	0.65

Contingency table showing the number of bees from LVG and HVG lines that self-groomed lightly and intensively within 3 min after placing 20 mg of flour on their thoraces, and the proportion of intense groomers from each genotype.

Compared to HVG, there were 64 down-regulated and 219 up-regulated DEGs in LVG worker brains with only 33 DEGs with a log2 FC ≥ 1 (**Table S3**). GO enrichment analysis showed 19 enriched pathways from a list of all 219 up-regulated genes (p<0.05; **Table 3**). The DEGs included the odorant binding proteins 16, 17, 18, and 21, and the gustatory receptor 10. The only enriched KEGG term (with up-regulated DEGs in LVG bees) was oxidative phosphorylation.

**Table 3:** GO enrichment analysis of cellular components and Kyoto Encyclopedia of Genes and Genomes (KEGG) terms.

Term name	Term ID	P adjusted value
Cytoplasm	GO:0005737	4.20×10^-3^
Envelope	GO:0031975	1.097×10^-4^
Inner mitochondrial membrane protein complex	GO:0098800	1.785×10^-4^
Intracellular membrane-bounded organelle	GO:0043231	1.838×10-2
Intracellular organelle	GO:0043229	2.297×10^-2^
Membrane-bounded organelle	GO:0043227	3.616×10^-2^
Membrane protein complex	GO:0098796	2.411×10^-4^
Mitochondrial envelope	GO:0005740	1.607×10^-5^
Mitochondrial inner membrane	GO:0005743	1.536x10^-5^
Mitochondrial membrane	GO:0031966	4.228x10^-5^
Mitochondrial protein-containing complex	GO:0098798	3.130x10^-3^
Mitochondrial proton-transporting ATP synthase complex	GO:0005753	1.030x10^-3^
Mitochondrion	GO:0005739	1.587x10^-5^
Organelle	GO:0043226	3.274×10^-2^
Organelle envelope	GO:0031967	1.097×10-4
Organelle inner membrane	GO:0019866	2.092x10^-5^
Organelle membrane	GO:0031090	1.290x10^-5^
Proton-transporting ATP synthase complex	GO:0045259	1.869×10-2
Oxidative phosphorylation	KEGG:00190	9.898×10^-8^

Significantly overrepresented terms based on DEGs in brain of LVG worker bees compared to HVG bees, using a cumulative hypergeometric test and multiple test corrections with g:SCS with adjusted p-values.

### 3.1 Identification of down-regulated DEGs and a biological pathway linked to high *V. destructor* population growth

We had an average of 127,693,996 ± 5,189,523 reads per sample (N=24) with an average of 105,674,348 ± 2,953,219 pseudo-aligning to the transcriptome. We found that the proportion of up and down-regulated genes in the different pairwise comparisons varied significantly ( $\chi_{(4, 250)}^{2}$ = 9.48, p=0.010). A *post-hoc* test showed significant differences in the proportion of DEGs in the pairwise comparisons of ‘LVG-light vs HVG-intense’ and ‘LVG-intense vs HVG light’ (p=0.03 and p=0.02, respectively). The proportion of up- and down-regulated DEGs for the ‘LVG-light vs HVG-intense’ category was of 0.036 and 0.084, respectively. Whereas the proportion of up- and down-regulated DEGs for the ‘LVG-intense vs HVG light’ comparison was of 0.024 and 0.29, respectively. The proportion of up-and down regulated DEGs in the pairwise comparisons ‘LVG-light vs HVG-light’, ‘LVG-intense vs HVG-intense’, and ‘HVG-light vs HVG-intense’ were not significantly different (p=0.31, p=0.37, and p=0.10; **Table S2**).

Compared to HVG, LVG honey bee brains showed significantly lower levels (p<0.05) of *Apis rhabdovirus-1* (log2 -5.96), VDV-1 (log2 -3.78)*, Apis rhabdovirus-2* (log2 -2.65), DWV-A (log2 -2.05), and *Varroa destructor virus-2* (VDV-2; log2 -0.72), but higher levels of *Sacbrood virus* (SBV; log2 6.59), *Apis filamentous virus* (log2 2.21), *Black queen cell virus* (BQCV; log2 1.86), *Acute bee paralysis virus* (ABPV; log2 1.80), IAPV (log2 1.55), and *Kashmir bee virus* (KBV; log2 0.99; **Table S4**). Also, from the 20 viruses included in the analysis, *Apis rhabdovirus 1*, *Apis rhabdovirus 2*, SBV, BQCV, DWV, and VDV-1 showed a proportion of positive samples ≥0.5, showing a possible high prevalence of these viruses in the tested population (**Table S4**). Additionally, DWV-A was negatively correlated with the expression of odorant binding proteins 16, 17, 18 and 21, and with the gustatory receptor 10 (R^2^=-0.46, -0.53, -0.55, -0.51, and -0.42, respectively; p<0.05). A positive correlation between time to first grooming and ABPV in HVG bees was observed (R^2 =^ 0.42, p=0.04).

### 3.2 Limited evidence of gene-expression patterns strictly associated with self-grooming intensity

We found no significant differences in the number of DEGs between light and intense groomers of the LVG genotype. Additionally, LVG-intense groomers bees showed lower levels of IAPV (log2 11.40), *Apis filamentous virus* (log2 11.29), KBV (log2 5.7), SBV (log2 1.63) and VDV-1 (log2 0.36), and higher levels of *Apis rhabdovirus 1* and *2* (log2 13.36 and 4.63, respectively), ABPV (log2 4.12), and BQCV (log2 0.48) compared to LVG-light groomers.

Compared to HVG-light, there were 8 up-regulated and 21 down-regulated DEGs in worker bee brains of the HVG-intense category (**Table S5**), including an up-regulation of protein lethal (2) essential for life, which has been linked to antiviral defense mechanisms in *Aedes aegypti* and *A. mellifera* (44, 45). The mean log2 fold change of the up-regulated DEGs was 1 ± 0 and 2.7 ± 0.62 for down-regulated DEGs; only 9 DEGs showed ≥ 1 fold log2 change, including peroxidase (**Table S5**). The GO enrichment analysis showed no significantly enriched pathways from a list of 29 DEGs (p<0.05). Additionally, HVG-intense bees had significantly higher levels of seven viruses (p<0.05): BQCV (log2 3.09), *Apis rhabdovirus 2* (log2 1.16), ABPV (log2 0.84), VDV-1 (log2 0.70), *Apis rhabdovirus 1* (log2 0.56), DWV-A (log2 0.51), and *Apis filamentous virus* (log2 0.27), but two viruses showed lower levels in HVG-intense bees (BQCV and VDV-2; log2 -9.67 and -1.85, respectively) compared to HVG-light bees.

### 3.3 A factor analysis finds an association between LVG and intense grooming, and a correlation between transcript expression and time of first grooming

A summary of the data using a factor analysis of mixed data showed an association between light grooming and HVG (Q1), and intense grooming with LVG (Q3; F1 and F2 explaining 68.87% of the variability). Odorant binding protein 17 (NM_001040207.1) contributed with 0.0018% to the model, and it was the only identified odorant binding protein in which a significant negative correlation was found between TPM and time of first grooming (**Table S6**; r=-0.376, p=0.07, α of 0.1). Odorant binding proteins 16, 18, 21 and the gustatory receptor 10 did not show a significant correlation between TPM and time of first grooming (*r*=-0.270, p=2.202; *r*=-0.292, p=0.16; *r*=-0.338, p=0.107, *r*=-0.289, p=0.176, α of 0.1, respectively). Also, a significant difference between the categories were found on TPM of the odorant binding protein 17 (F_(43,120)_=3.682, p=0.029, R^2 =^ 0.356); odorant binding protein 17 was double in LVG bees compared to HVG bees, there was no difference between light and intense groomers, and no interaction between varroa growth and grooming intensity was found (p>0.05).

## 4 Discussion

Results of this study showed that intense grooming may not accurately be used to differentiate LVG and HVG bees since both genotypes had higher proportions of intense groomers than light groomers. Although the method to identify light and intense groomers have been used in previous studies (26, 28), a more accurate and automated method to identify different parameters associated with light and intense self-grooming behavior should be further explored. Still, time of first grooming seems to be a more informative variable to differentiate bees selected for LVG and HVG. Differences in time of first grooming between honey bee genotypes that are presumably resistant and susceptible to *V. destructor* were previously noted (28), but it appears that bees performing intense and light self-grooming also differ for the time of first response to the irritant. In this study, LVG-intense and HVG-intense groomers responded faster to the irritant compared to light-groomers of both genotypes, with LVG-intense groomers being faster to respond to the irritant. However, a consideration of the main effect of grooming intensity, and not the genotype, based on ART should be considered; it is possible that more marked effects will be seen in the next generations of the selective breeding. Additionally, although only one experienced observer conducted the assessments, this study failed to account for the possible bias of the observer. Thus, future studies on behavioral immune responses should consider the influence that a single observer could have in the assessments, or the interobserver effect if two or more observers are classifying the behavior (46, 47). These results support the notion that both grooming behavior and resistance to *V. destructor* may be associated with bee sensitivity and speed of reaction. This inference is also supported by a previous study that identified quantitative trait loci (QTL) associated with fast grooming bees of two genotypes (48). Furthermore, a recent study found that bees that were individually exposed to *V. destructor* parasitism stung significantly faster than bees not exposed to the mite (49), suggesting that the irritation caused by the mite influenced the speed of response of the experimental bees. Thus, it appears that sensitivity to an irritant and speed of reaction are mechanisms that allow bees to efficiently get rid of the irritant and prevent parasitism. Further studies are needed to confirm the effect of different irritants, like flour or mites, on the speed of motor reactions and their consequences on colony fitness. Furthermore, the degree of sensitivity of bees to an irritant may affect their grooming response threshold, as it appears to be the case of LVG and HVG bees for time to first grooming. The fast perception of the irritant results in triggering motor responses expressed as self-grooming. Interestingly, four odorant binding proteins were up-regulated in LVG bee brains compared to HVG bees, which have been previously associated with behavioral responses against *V. destructor* (50). Additionally, six compounds were identified in *V. destructor* mites triggering the behavioral immune response of hygienic behavior in worker bees, including tricosan-2-one, pentacosan-2-, one, and tetracosyl acetate (51), indicating that chemical cues can be involved in the perception of parasites by the host and activate defense mechanisms. Furthermore, bees performing Varroa Sensitive Hygiene (VSH), a form of hygienic behavior triggered by *V. destructor* (52), showed 11 differentially expressed genes in their antennae related to olfactory functions, including the up-regulation of *odorant binding protein 3* (53). The odorant binding protein 3 was found down-regulated in the brain of VSH bees. Still, it appears that olfactory receptors expressed in non-sensory organs of insects are not directly associated with odor perception, but they are involved in many other functions, including anti-inflammatory processes (54). Moreover, odorant binding proteins are essential for the performance of important insect behaviors, like reproduction, feeding, and developmental processes (55, 56). The expression of odorant binding proteins genes 3, 16, 17, 18, 19, 20, and 21 has been detected in the brains of honey bees, indicating that the function of these genes in highly specialized tissues could be linked to different biological processes, yet to be investigated (56). The expression of odorant binding proteins in brains of other animal models, such as *Mus musculus* and *Drosophila* spp, has also been reported with evidence of dysregulation linked to neurodegenerative diseases, including chronic schizophrenia (57, 58). Interestingly, olfactory dysfunction in patience with schizophrenia has been documented (58, 59). Hence, the effect of odorant binding proteins 16, 17, 18 and 21 on the ability of the bees to perform neural processes relevant to behavioral immune responses, such as self-grooming, should be further investigated. Special attention could be paid to odorant binding protein 17, as its expression was negatively correlated with the time of first response to the irritant, indicating that high levels of its expression could be related to a faster response to the irritant and consequently lower levels of varroa mites. However, it would be interesting to correlate other quantitative traits linked to grooming behavior to transcript or gene levels, as there could be other informative traits linked to the control of *V. destructor*. Additionally, the negative correlation in LVG bees between DWV-A and the expression of odorant binding proteins 16, 17, 18 and 21 could be related to the lesser effect of the neural DWV-A infection on neural processes related to self-grooming, and possibly mediated by odorant binding proteins. Effects of DWV-A on cognitive process, like memory retention, have been documented, including learning and memory (60), but to the best of our knowledge there are no studies on the effect of neurotropic viruses on behavioral immune responses. Although Mondet et al. (53) found higher levels of DWV in the antennae of VSH bees, the relationship between mite levels and viral levels *via* the control of the parasite through VSH behavior should be considered. The expression of gustatory receptors in the honey bee brain and their involvement in sensory response and social organization has been described (61), indicating that gustatory receptor genes could be linked to complex biological functions. Thus, the role of the odorant binding proteins 16, 17, 18 and 21, and the gustatory receptor 10 in neural processes relate to the performance of self-grooming should be investigated, as they could be crucial molecules for the execution of defense mechanisms.

There seems to be an association between grooming and defensive behavior of honey bees. A study found a correlation between defensive behavior, measured as the number of bees recruited in response to alarm pheromone (isopentyl acetate), and the proportion of injured mites in honey bee colonies (62), but the study did not determine if the phenotypic correlation had a genetic component. Another study reported that an inhibition of the oxidative phosphorylation pathway increased aggression in honey bees (63). Thus, self-grooming and defensive behavior might share common molecular mechanism associated with energy metabolism in the brain. The perception of the irritant or the perception of alarm pheromone could be triggering similar neural responses that require an additional production of energy through oxidative phosphorylation or glycolysis (63, 64). This study found an inverse pattern to defensive behavioral responses since up-regulated DEGs were linked to oxidative phosphorylation in LVG bees. Hence, predicting differences in behavioral immune responses between honey bee colonies selected for LVG and HVG could be possible if both behaviors (self-grooming and defensive behavior) share similar mechanisms. The LVG and HVG bees used in this study were not assessed for defensive behavior. Thus, future studies aimed at comparing self-grooming and defensive behavior in LVG and HVG bees would be needed to confirm if both behaviors share similar biological processes. Additionally, the down-regulation of genes associated with oxidative phosphorylation in HVG bees could be an effect of the feeding behavior of *V. destructor*. The mites feed primarily on fat body tissue (65), which has the functions of storing and releasing energy (66). However, when comparing the down-regulated DEGs found in this study to 78 down-regulated DEGs found by Morfin et al. (27) in the brains of bees also subjected to self-grooming assays and parasitized by *V. destructor* in laboratory conditions, we found only two DEGs in common (GB45073 and GB42468). If an overlap of DEGs between the dataset reported by Morfin et al. (27) and the one reported in this study had been found, the DEGs would have been linked to the effect of the parasitosis by *V. destructor* and not the genotype. Also, an analysis using their 78 down-regulated DEGs showed another enriched energy metabolic KEGG pathway, starch and sucrose metabolism. It is possible that *V. destructor* is affecting energy metabolism, but LVG bees are able to increase energy resources through oxidative phosphorylation to trigger self-grooming instances against the mites. Hence, future studies on the DNA profiling of LVG and HVG bees will be helpful to identify genotypic differences and confirm the use of the identified DEGs as molecular markers for selective breeding. In addition to the above, no significant differences in the number of DEGs between light and intense groomers of the LVG genotype were found. It is possible that the quick response of LVG bees to the irritant did not allow to perceive significant changes in gene expression. Additional studies exploring gene expression differences between HVG and LVG colonies would benefit from a common colony approach to completely eliminate the possibility of *V. destructor* infestation leading to variance in gene expression across conditions.

The significant lower levels of DWV-A in LVG bees align with previous studies (27), indicating that traits associated with LVG could be linked to the restraining of some viral infections, like DWV-A and VDV-1, perhaps through the control of their biological vector, *V. destructor*. However, the significantly higher levels of *Apis rhabdovirus-2* in LVG-intense groomers compared to light groomers and the lower levels of BQCV and VDV-2 in HVG-intense bees, demonstrates the importance of investigating its potential pathogenicity and impact on bee health, including the regulation of immune responses in the central nervous system (67). Also, studying the potential neurotropism of viruses and their role in behavioral impairment should be confirmed. For example, studies have found that neurotropic viruses, like the *Feline immunodeficiency virus*, can cause neurodegeneration leading to behavioral and neurophysiological impairment in its host (68). Viral infections, such as ABPV and KBV, possibly connected to *V. destructor* parasitism (69), could be impacting the neural ability of the bees to perceive the irritant and react to it effectively, hindering behavioral immune responses, such as self-grooming. ABPV is a neurotropic virus characterized by inflicting motor impairment in the infected host (69). This study found a positive correlation between time of first grooming and ABPV levels in HVG bees, suggesting a possible effect of the viral infection in the ability of the bees to perform self-grooming. In addition to the above, exploring viral dynamics taking into consideration effects of individual defense mechanisms should be explored, as proposed by Piot and Smagghe (70), but also the effects of behavioral immune responses as it could provide more information about the impact of viruses, vectored and not vectored by *V. destructor* (like BQCV and *Apis rhabdovirus-2*), on honey bee health. This results not only emphasize the possible impact of viral infections on behavioral immune responses, but also that breeding programs should use genomic assisted selection to avoid the interference of stressors in the performance evaluations, such as *V. destructor* parasitism and viral infections. The results of this study highlight differences between bees from LVG and HVG selected colonies and reports on realistic outcomes on gene expression analysis and viral levels, but further studies focused on genotyping LVG and HVG selected bees should be able to confirm possible molecular markers, such as odorant binding proteins, to assist breeders.

The results of this study could serve as basis of future evaluations of the next generations of LVG and HVG selected bees, and may advance the path for selecting honey bee stocks resistant to *V. destructor* (and associated viruses) using genomic tools. Viral infections in the central nervous system should be investigated to determine their impact on neural processes that regulate behavioral immune responses such as grooming intensity and time of first response to irritants.

## 5 Conclusions

This study showed the possible involvement of odorant binding protein genes in the perception of irritants, which would trigger rapid self-grooming instances in bees, and the activation of energy metabolic pathways. Our results provide novel information on the molecular mechanisms of behavioral immune responses in bees from colonies selected for LVG and HVG. Differences in viral levels between the brains of LVG and HVG bees showed the importance of investigating the pathogenicity and potential impacts of neurotropic viruses on behavioral immunity. Taken together, the results of this study advance the understanding of a trait used for selective breeding and emphasizes the need of genomic assisted selection tools to prevent selecting for traits sensible to stressors.

## Data availability statement

The datasets generated for this study can be found in Dryad repository https://doi.org/10.5061/dryad.59zw3r29q and NCBI, accession number PRJNA938182.

## Author contributions

Conceptualization: NM, BH, EG-N. Methodology: NM, BH, EG-N. Investigation: NM, BH, EG-N, AM. Visualization: NM, BH. Writing—original draft: NM, BH. Writing—review & editing: NM, BH, AM, EG-N. All authors contributed to the article and approved the submitted version.

## Supplementary material

The Supplementary Material for this article can be found online at: https://www.frontiersin.org/articles/10.3389/finsc.2023.951447/full#supplementary-material and Dryad.
